# The impact of infarct size on regional and global left ventricular systolic function: a cardiac magnetic resonance imaging study

**DOI:** 10.1186/1532-429X-17-S1-P121

**Published:** 2015-02-03

**Authors:** Alberto Palazzuoli, Matteo Beltrami, Chiara Bucciarelli-Ducci, Armadeep Gosh Dastidar, Ranuccio Nuti, Gianni D Angelini

**Affiliations:** Internal Medicine UO Cardiovascular diseases, Univesity of Siena, Le Scotte hospital, Siena, Italy; Bristol Heart Institute, University of Bristol, Bristol, UK

## Background

Myocardial infarction results in myocardial scarring which can have an impact on left ventricular (LV) stiffness and contractile function, ultimately leading to reduced LV systolic function and LV remodelling, However controversy remains about the relation between scar extension and segmental wall motion contractility global systolic function, and LV remodelling.

To elucidate the relationship between scar extension, wall motion score index (WMSI), LV dimensions and systolic function in a group of patients with previous myocardial infarction by cardiac magnetic resonance (CMR).

## Methods

133 patients with previous (>6 month) myocardial infarction were retrospectively enrolled in the study. Indexed end-systolic volume (ESVi), indexed end-diastolic volume (EDVi), LV ejection fraction (EF), stroke volume (SV), left ventricular mass, WMSI and sum scar score (SSS) were measured using cardiac magnetic resonance.

## Results

A total of 2261 segments were studied: regional wall motion abnormalities were present in 1032 segments (45 %) and 724 (32 %) showed presence of myocardial infarction (late myocardial enhancement) and segments. WMSI correlated significantly with EF (r=-0.87 p<0.0001) in all patients; both in patients with EF ≥40% (r=-0.77 p<0.0001) than in patients with EF<40% (r=-0.68 p<0.0001). WMSI also correlated significantly with SSS (r=0.57, p<0.0001). However, correlation between WMSI and SSS was more significant in patients with transmural myocardial infarction (WMSI 2.1±0.5 and SSS 17±8; r=0.5 p<0.0001) than non transmural myocardial infarction (WMSI 1.6±0.7 and SSS 6±4; r=0.3 and p=0.02). Significant correlation was found between EF and SSS (r=-0.55 and p<0.0001) and between SSS and LV indexed volumes (EDVi; r=0.44, p<0.0001 and ESVi; r=0.51, p<0.0001).

## Conclusions

Patients with transmural MI demonstrated a more impaired global and regional systolic function together with increased LV dimension. Beyond transmurality, scar extension evidenced even more relation with both segmental and global wall motion abnormalities as well as systolic dysfunction. This emphasised the importance of CMR to evaluate all at once scar dimensions, segmental wall motion and subsequent LV remodelling.

## Funding

This research was supported by NIHR Bristol Cardiovascular Biomedical Research Unit.

